# Pleiotropic effects of BET inhibition broadly boost tumor immunogenicity to CD8^**+**^ T cells

**DOI:** 10.1080/2162402X.2026.2658916

**Published:** 2026-04-29

**Authors:** Jeroen Melief, Lucas Baldran-Groves, Marc-Antoine Gerault, Ying Yu Liang, Sylvya Pasca, Mireia Cruz de los Santos, Stina Wickström, Tanja Lövgren, Anderson Daniel Ramos, Lars-Gunnar Larsson, Pär Nordlund, Barbara Seliger, Rolf Kiessling

**Affiliations:** a Department of Oncology-Pathology, Karolinska Institutet, Stockholm, Sweden; b Department of Oncology-Pathology, Theme Cancer, Patient Area Head and Neck, Lung and Skin, Karolinska University Hospital, Stockholm, Sweden; c Department of Immunology, Genetics and Pathology, Uppsala University, Uppsala, Sweden; d Department of Pharmaceutical Biosciences, Uppsala University, Uppsala, Sweden; e Institute of Translational Immunology, Faculty of Health Sciences, Brandenburg Medical School, Brandenburg an der Havel, Germany; f Institute of Pathology, Martin Luther University Halle-Wittenberg, Halle (Saale), Germany; g Fraunhofer Institute for Cell Therapy and Immunology, Leipzig, Germany

**Keywords:** Cancer immunotherapy, bromodomain and extra-terminal inhibitors, tumor-infiltrating lymphocytes, tumor immunogenicity, immune escape

## Abstract

BET inhibitors (BETi) have shown potential to augment tumor immunogenicity in melanoma. However, conflicting evidence exists regarding their precise mechanism of action, and their overall impact on melanoma immunogenicity and antitumoral T cell responses remains unclear. To address this, human melanoma cell lines treated with JQ1 and/or IFNγ were investigated for gene and protein expression changes in key pathways governing immunogenicity and cocultured with autologous tumor-infiltrating lymphocytes (TIL) with known antigen-specificity. JQ1-induced proteome-wide alterations were examined using mass spectrometry-based cellular thermal shift assay (MS-CETSA), which revealed that JQ1 broadly impacts melanoma immunogenicity by regulating IFN signaling, antigen processing and presentation, and innate immune signaling pathways. More specifically, JQ1 enhanced JAK1/STAT1 signaling and upregulated components of the HLA class I (HLA-I) antigen processing and presentation machinery (APM), increased MART-1 expression while concomitantly dampening tumoral expression of PD-L1, IDO1, and HLA class II (HLA-II). Functionally, JQ1 markedly improved tumor recognition by autologous MART-1- and neoantigen-specific CD8^+^ TIL, while dampening CD4^+^ TIL activation through the downregulation of Cathepsin S (CTSS). Preliminary results using JQ1-treated melanoma cells in a mixed lymphocyte-tumor cell culture (MLTC) markedly enhanced TIL proliferation and resulted in a T cell product enriched for CD8^+^ T cells. These findings reveal how the pleiotropic effects of BETi on melanoma cells broadly boost their immunogenicity towards CD8^+^ T cells and uncover novel pathways that might be therapeutically exploited to enhance CD8^+^ T cell-mediated anti-tumor immunity in *ex vivo* and *in vivo* approaches to cancer immunotherapy.

## Graphical abstract

**Figure:**
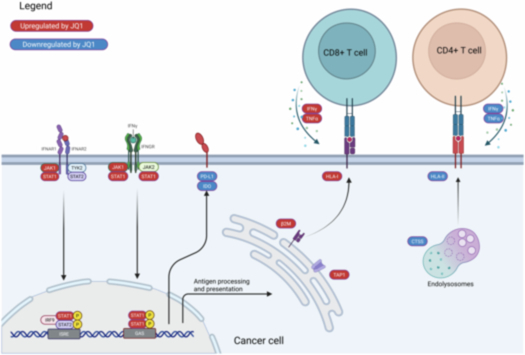

## Background

While the treatment of cancer with immune checkpoint blockade (ICB) or T cell-based adoptive cell therapies (ACT) has dramatically improved the survival of cancer patients, these approaches still fail to induce durable clinical benefit due to primary or delayed immune resistance.^1^^,^^2^ Poor tumor immunogenicity is a well-recognized root cause of immune resistance and is frequently attributed to disruptions in interferon (IFN) signaling, the HLA class I (HLA-I) antigen processing and presentation machinery (APM), or to inflammation-induced dedifferentiation.^3-6^ Therefore, strategies aimed at enhancing tumor immunogenicity hold significant potential to substantially improve cancer immunotherapy outcomes.

Bromodomain and extraterminal domain (BET) proteins, which comprise BRD2, BRD3, BRD4, and BRDT, are epigenetic readers that bind acetylated histone lysines and recruit regulators of transcriptional programs that control cell identity, oncogenic signaling, and inflammatory responses. By coordinating chromatin accessibility and transcriptional responses to cytokine signaling, BET proteins can profoundly influence the molecular mechanisms that govern tumor immunogenicity and immune recognition.^7^ Pharmacological inhibition of BET bromodomains using small-molecule BET inhibitors (BETi) has therefore emerged as a strategy to modulate BET-dependent transcriptional programs in cancer cells and potentiate anti-tumor immunity.^8-12^ However, conflicting evidence exists, as it has also been reported that BET inhibition can suppress the therapeutic effects of PD-1 blockade in the B16F10 melanoma mouse model.^13^ Thus, the extent to which BET-regulated transcriptional programs influence melanoma immunogenicity, the molecular pathways driving these effects, and their ultimate impact on immune recognition by human tumor-specific T cells remain largely undefined. Therefore, we used JQ1, a well-characterized competitive BETi, to investigate in detail how BET inhibition affects pathways governing tumor immunogenicity and subsequently influences functional recognition by autologous human tumor-infiltrating lymphocytes (TIL). We show that JQ1, both by itself and in combination with IFNγ, sensitizes human melanoma cells for CD8^+^ TIL recognition across multiple types of cancer antigens. Using mass spectrometry–cellular thermal shift assay, we characterized the proteome-wide effects of JQ1 on IFNγ-inducible determinants of tumor immunogenicity, which uncovered novel tumor-intrinsic pathways governing both anti-tumoral CD8^+^ and CD4^+^ T-cell responses. Finally, our study offers early evidence that BETi-treated tumor cells can serve as an effective stimulus for the selective activation and *ex vivo* expansion of tumor-specific CD8^+^ T cells, which points toward the potential utility of BETi in enhancing adoptive T cell therapies in melanoma and perhaps other solid cancers.

## Methods

### Tumor cell culture and treatment

Early-passage melanoma cell lines ANRU, ROAL, and KADA were established from metastatic melanoma lesions at the Clinic of Oncology, Karolinska University Hospital, according to a previously published protocol.^14^ Experiments were conducted between 2016 and 2026 at the Department of Oncology–Pathology, Karolinska Institutet. ANRU and ROAL melanoma cells were cultured in IMDM, while A375, KADA (melanoma), HCT116 (colorectal cancer), HeLa (cervical cancer), and U2946 (B-cell lymphoma) cells were cultured in RPMI. A549 (non-small cell lung cancer) cells were cultured in DMEM (all media from Gibco, Life Technologies). All media were supplemented with 10%–20% fetal bovine serum and 1% penicillin–streptomycin. Unless otherwise indicated, the cells were treated for 72 h with IFNγ (25 ng/mL), IFNα (10 ng/mL), and/or JQ1 (0.4 µM). DMSO-treated cells served as controls.

### TIL culture and tumor/TIL cocultures

The culture and expansion of TIL from patient ANRU, KADA, and ROAL, as well as the sorting of antigen-specific T cell populations, was done as published before.^14^^,^^15^ CD8⁺ and CD4⁺ TIL were isolated using Microbeads and LS columns (Miltenyi). Autologous TIL recognition of untreated or JQ1/IFNγ-treated tumor cells was assessed at a 5:1 effector:target ratio. Where indicated, HLA-I was blocked with 20 μg/mL anti-HLA-ABC (W6/32, BioLegend) for 30 min at 37 °C. TIL activated with CD3/CD28 Dynabeads (Life Technologies) served as a positive control. For flow cytometry analysis, cocultures were incubated 1 h with anti-CD107a (BioLegend), followed by 3 h with GolgiPlug/GolgiStop (BD) before staining. For MART-1 dextramer assays, TIL were incubated for 10 min at room temperature with MART-1 dextramers (Immudex) prior to extracellular staining.

### IFNγ ELISA

Sandwich ELISA was performed to detect secreted IFNγ in the supernatant of tumor-TIL coculture plates. IFNγ ELISAs (Mabtech) were done following the manufacturer's protocol. Analysis was done in GraphPad Prism software (version 10).

### Expression analysis by qPCR

Tumor cells were treated with JQ1 (0.4 µM) and/or IFNγ for 72 h. Cells were washed with PBS, and total RNA was extracted using TRIzol and purified (PureLink RNA Mini Kit). cDNA was synthesized from 500 ng RNA (iScript kit). qPCR was performed with SYBR Green on a QuantStudio 7 Flex system. Primers used in this study are displayed in Table 1. Relative changes in gene expression were quantified by the ΔΔCT method, normalized to the housekeeping gene β-actin.

**Table 1.** Primer list for RT-qPCR.

Primer	Sequence
HLA-I heavy chain-forward	GCC TAC CAC GGC AAG GAT TAC
HLA-I heavy chain-reverse	GGT GGC CTC ATG GTC AGA GA
β2M-forward	CTC GCG CTA CTC TCT CTT
β2M-reverse	AAG ACC AGT CCT TGC TGA
TAP1-forward	GGA ATC TCT GGC AAA GTC CA
TAP1-reverse	TGG GTG AAC TGC ATC TGG TA
TAP2-forward	CCA AGA CGT CTC CTT TGC AT
TAP2-reverse	TTC ATC CAG CAG CAC CTG TC
TAPASIN-forward	TGG GTA AGG GAC ATC TGC TC
TAPASIN-reverse	ACC TGT CCT TGC AGG TAT GG
LMP2-forward	TGC TGC ATC CAC ATA ACC AT
LMP2-reverse	TGT GCA CTC TCT GGT TCA GC
LMP7-forward	TCT GCG TCA TCA GCA AGA AC
LMP7-reverse	GCC ATT CAG GAA GTG TCC AT
LMP10-forward	GGG CTT CTC CTT CGA GAA CT
LMP10-reverse	CAG CCC CAC AGC AGT AGA TT
CIITA-forward	AGA AGT TCC TCG GAA GAC ACA G
CIITA-reverse	TGT TGT TCT GGG ACA GAT TGA G
JAK1-forward	GCA CCA TCA CCG TTG ATG AC
JAK1-reverse	TCC AGT GAG CTG GCA TCA AG
STAT1-forward	ATC CTC GAG AGC TGT CTA
STAT1-reverse	GCC AGG TAC TGT CTG ATT
CTSS-forward	TGTAGATGCGCGTCATCCTTC
CTSS-reverse	CCAACCACAAGTACACCATGAT
β-actin-forward	TCC TGT GGC ATC CAC GAA ACT
β-actin-reverse	GAA GCA TTT GCG GTG GAC GAT

### Western blot

Treated cells were lysed in IP buffer with protease inhibitors, and protein concentration was measured by BCA assay (Thermo Fisher). Proteins were separated by SDS–PAGE, transferred to nitrocellulose, blocked with 5% BSA, and incubated with primary antibodies overnight at 4 °C: phospho-Jak1 (3331S, Cell Signaling Technology) and phospho-STAT1 (9167S, Cell Signaling Technology). Membranes were probed with HRP- or IRDye-conjugated secondary antibodies and detected using ECL (iBright) or LI-COR Odyssey. Bands were quantified using QuantityOne and normalized to GAPDH (sc-47724, Santa Cruz Biotechnology).

### Flow cytometry

Antibodies used for flow cytometry are listed in Table 2. Treated tumor cells or TIL were harvested, washed, and incubated with a dead cell marker diluted in PBS for 15 min at room temperature. For extracellular stainings, cells were incubated with antibodies for 30 min at 4 °C. For intracytoplasmic and intranuclear staining, cells were fixed for 30 min at 4 °C using BD Cytofix/Cytoperm™ Fixation/Permeabilization Kit or eBioscience™ Intracellular Fixation & Permeabilization Buffer Set according to the manufacturer's instructions. Antibodies used for intracellular staining were diluted in kit wash buffers and incubated for 30 min at 4 °C. All samples were acquired on a Novocyte 3000 or Novocyte Quanteon flow cytometer (Acea Biosciences, San Diego, CA, USA) and data analyzed using FlowJo software v10 (Treestar). Gating strategies for tumor and T cells are shown in Supplementary Figure S1.

**Table 2.** List of antibodies used for flow cytometry.

Antibody/dye	Label	Manufacturer	Catalog
Dead cell marker	Aqua	Thermo Fisher Scientific	L34966
	eFluor660	eBioscience	65-0864-14
Anti-human HLA-A, B, C	APC/Cy7	BioLegend	311426
Anti-human HLA-DP, DQ, DR	FITC	BioLegend	361706
Anti-human PD-L1	BV786	BD	563739
Anti-human IDO	PE-Cy7	eBioscience	25-9477-42
Anti-human MART-1	PE	Santa Cruz Biotechnology	sc-20032 PE
Anti-human NGFR	PerCP/Cy5.5	BioLegend	345112
Anti-human CD3	PE/Cy7	BioLegend	300420
Anti-human CD8	APC/Cy7	BioLegend	344714
Anti-human CD4	PerCP	BioLegend	317432
Anti-human IFNγ	PE	BioLegend	506507
Anti-human TNFα	BV785	BioLegend	502948
Anti-human Ki-67	BV421	Nordic Biosite	350506

### Cellular thermal shift assay (CETSA)

ANRU cells were seeded in T25 flasks and allowed to adhere overnight before treatment for 24 h with DMSO, IFNγ (25 ng/mL), JQ1 (0.4 µM), or JQ1 in combination with IFNγ. Three biological replicates were included for each condition. Cells were harvested by brief trypsinization, resuspended in HBSS supplemented with protease inhibitors, and pelleted by centrifugation. After resuspension in 300 µL HBSS, 100 µL of cell suspension per condition was distributed into PCR tubes and heated at 37 °C, 52 °C, or 58 °C for 3 min using a thermocycler, followed by cooling at 4 °C for 3 min. Cells were lysed by three freeze–thaw cycles in liquid nitrogen and centrifuged at 20,000 × *g* for 20 min at 4 °C. Supernatants (80–90 µL) were collected and stored at −80 °C until mass spectrometry analysis.

#### Sample preparation for mass spectrometry

Protein concentrations were measured using the Pierce™ BCA Assay Kit (Thermo Scientific), and equal amounts were processed for proteomics. Samples were reduced with TCEP, alkylated with 2-chloroacetamide, and digested sequentially with Lys-C and SOLu-Trypsin. Peptides were desalted, labeled with 10-plex TMT, quenched with TFA, pooled, and fractionated by high-pH reverse-phase LC into 12 fractions for LC–MS analysis.

#### LC–MS analysis

Peptide fractions were resuspended in 0.1% formic acid and analyzed on a Dionex UltiMate 3000 UPLC coupled to a Q Exactive HF (Thermo Scientific) using a 50 cm × 75 µm C18 column and a 70-min gradient. Data were acquired in top-12 DDA mode, with full MS scans at 70,000 resolution (375–1500 m/z) and MS/MS scans at 70,000 resolution with a 1.2 m/z isolation window.

#### Protein identification and quantification

Proteins were identified using Proteome Discoverer v3.1 with Sequest HT against the reviewed human UniProt database and rescored with INFERYS. Tolerances were 10 ppm (precursor) and 0.02 Da (fragment), allowing two missed cleavages. Oxidation (M) was dynamic; carbamidomethylation (C) and TMT10plex (K, N-term) were static. Spectra with S/N > 1.5 were used, and FDR was 1% at peptide and protein levels. Quantification used unique and razor peptides; only master proteins were retained. Data were processed in R with IMPRINTS.CETSA, including normalization, log2 fold-change calculation, and removal of contaminants, missing values, or low-abundance proteins.

#### Differential analysis

Differential analysis was performed using the imprints_IS function from the IMPRINTS.CETSA.app package with an FDR threshold of 5% and a minimum |I-score| ≥ 1.5. Protein hits were defined as those displaying at least one |I-score| ≥ 1.5 in the following comparisons: JQ1 vs DMSO, IFNγ vs DMSO, or JQ1 + IFNγ vs DMSO. I-scores were subsequently recomputed for the final hit list without z-score normalization to better reflect log2 fold-change values of the IMPRINTS-CETSA profiles.

To assess the effect of the combined treatment, the I-score ratio between the JQ1 + IFNγ combination and IFNγ alone was calculated. Repeated-measures ANOVA was performed for each protein to evaluate treatment effects on fold changes. Proteins with I-scores > 0.25 were classified as upregulated and those with I-scores < −0.25 as downregulated. Proteins with ANOVA *p* < 0.05 and an I-score ratio > 1 were categorized as Amplified (if IFNγ I-score was positive) or Lowered (if negative), whereas proteins with an I-score ratio < 1 were categorized inversely.

To further characterize changes in protein stability and/or abundance, proteins were also classified into CC, CN, NC, and NN categories using the imprints_IS function as previously described.^16^^,^^17^

#### Protein-protein interaction network from MS-CETSA data

A protein–protein interaction network was generated using the STRING app in Cytoscape (confidence ≥ 0.4). Nodes represent proteins, edges indicate interactions, fill colors show IFNγ effect, and node colors reflect JQ1 + IFNγ versus IFNγ effect.

### RNA interference

Silencing select RNAs targeting MART-1 (4392420, ID: s5272, ThermoFisher), CTSS (cat. 4392420, ID: s3764, ThermoFisher), or non-targeting control silencing RNAs (cat. 4390843, ThermoFisher) were transfected using Lipofectamine RNAiMAX (cat. 13778075, ThermoFisher) according to the manufacturer's instructions.

### Mixed Lymphocyte-Tumor cell culture (MLTC)

MLTC experiments were conducted as previously established.^18^ Briefly, 2 × 10^6^ ANRU tumor cells were seeded in T75 flasks and treated with DMSO or JQ1 (0.4 µM) for 72 h. Cells were harvested, irradiated (100 Gy), and cocultured with autologous ANRU TIL in 24-well plates at an E:T ratio of 10:1 in AIM V medium (Gibco) supplemented with IL-4 (5 ng/ml, Cellgenix) and low-dose IL-2 (150 IU, PeproTech). Culture medium containing IL-2 was refreshed every 2/3d. TIL were cultured for a total of 19 d and supplemented weekly with irradiated tumor cells treated as described above (3 times in total).

### Statistical analysis

Unless stated otherwise, statistical analysis was performed in GraphPad Prism (v10). Data is represented as mean ± SD of three or more independent experiments unless stated otherwise. Repeated measures one-way or two-way ANOVA and post-hoc Holm-Šídák multiple comparison tests were used to test differences between conditions. Significant *p-*values are annotated accordingly: *p < 0.05; **p < 0.01; ***p < 0.001; and ****p < 0.0001.

## Results

### Pretreatment of tumor cells with JQ1 boosts activation of cocultured autologous CD8^+^ TIL and dampens CD4^+^ T cell responses

**Figure 1.**
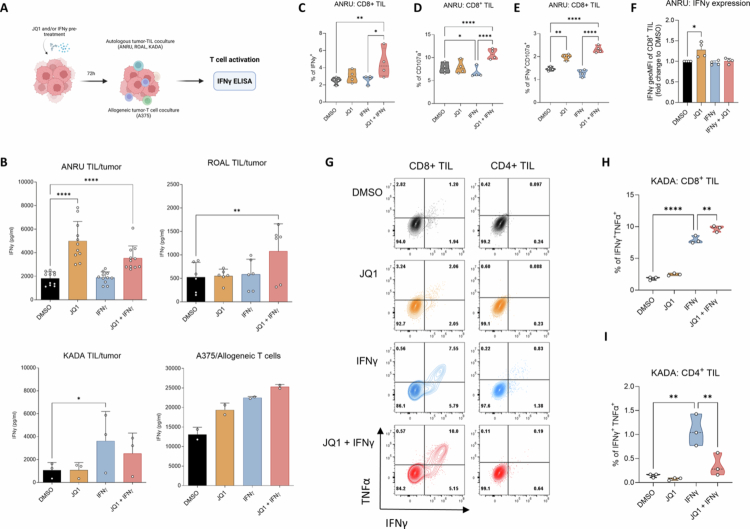
Pretreatment of tumor cells with JQ1 boosts the activation of autologous CD8^+^ TIL while dampening activation of CD4^+^ T cells. (A). Schematic overview of coculture experiments. (B). Recognition of tumor cells pretreated with IFNγ, JQ1 (0.4 μM), or the combination of both for 72 h by autologous TIL (ANRU, ROAL, and KADA, *n* ≥ 3 biological replicates), or allogeneic T cells from healthy donor blood (A375, *n* = 2 technical replicates), as assessed by secreted IFNγ after 24 h coculture. (C). Frequency of IFNy^+^, (D). CD107a^+^ or (E). IFNy^+^CD107a^+^ ANRU TIL gated on CD3^+^CD8^+^ T cells after coculture with treated autologous tumor cells. (F). Mean IFNy production by CD8^+^IFNy^+^ ANRU TIL upon coculture with treated tumor cells. (G). Representative flow cytometry plots of IFNy producing and/or TNFα producing CD8^+^ (left) or CD4^+^ KADA TIL upon coculture with treated autologous tumor cells. (H and I). JQ1 augments cytokine production of CD8^+^ T cells and dampens IFNγ-induced CD4^+^ T cell activation, as assessed by frequencies of IFNγ^+^TNFα^+^ T cells upon coculture of KADA TIL with JQ1, IFNγ, or JQ1 + IFNγ treated autologous tumor cells (*n* = 3).

To investigate the impact of BET inhibition on tumor immunogenicity, melanoma cell lines ANRU, ROAL, and KADA were pretreated with the BETi JQ1, either alone or in combination with IFNγ, and subsequently cocultured for 24 h with autologous TIL; similarly pretreated A375 cells were cocultured with allogeneic T cells from healthy donors (Figure 1A). We studied the time kinetics of the pretreatment effects in ANRU, along with effects on tumor cell viability. Sub-apoptotic doses of JQ1 enhanced recognition by ANRU TIL upon 48 to 72 h tumor cell pretreatment in an HLA-I-dependent manner, as blockade of HLA-I abrogated IFNγ release by TIL (Supplementary Figure S2A–C). In all tumor cell lines, apart from KADA, 72 h pretreatment with JQ1, either alone or in combination with IFNγ, led to improved recognition by bulk TIL and allogeneic T cells, as indicated by secreted IFNγ levels in the coculture supernatants (Figure 1B). Notably, 72 h pretreatment of tumor cells with IFNγ alone only improved T cell-mediated recognition of KADA and A375 cells, but not of ANRU and ROAL cells. Flow cytometry analysis confirmed these findings in ANRU, where pretreatment with JQ1 alone or in combination with IFNγ increased the frequency of IFNγ-producing (IFNγ^+^) and/or degranulating (CD107a^+^) CD8^+^ TIL, as well as IFNγ production by CD8^+^ T cells (Figure 1C–F). Interestingly, ANRU TIL were comprised of a population of CD4^+^ T cells (~20% of CD3^+^ TIL) that were largely non-responsive to untreated or treated autologous tumor cells (data not shown). In KADA, however, JQ1 treatment enhanced IFNγ-induced activation of CD8^+^ T cells while impairing cytokine production by CD4^+^ TIL (Figure 1G–I). Thus, JQ1 selectively improves the effector functioning and activation of autologous CD8^+^ T cells in an HLA-I-dependent manner while suppressing CD4^+^ T cell responses.

### Proteome-wide analysis reveals that JQ1 regulates tumor immunogenicity through pleiotropic effects

**Figure 2.**
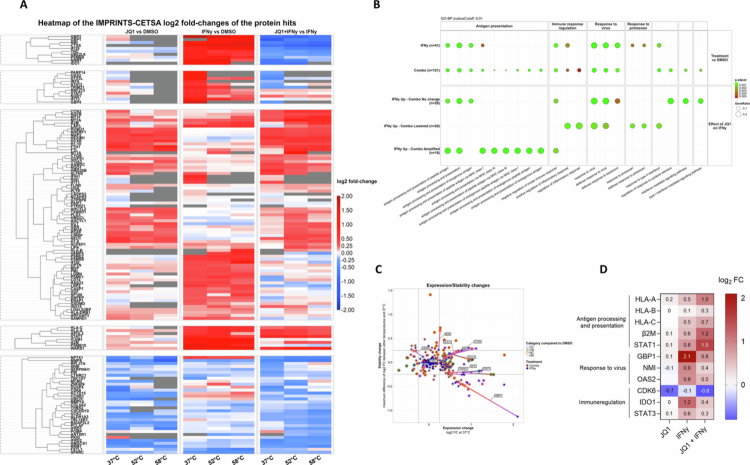
Proteome-wide analysis reveals that JQ1 regulates tumor immunogenicity through pleiotropic effects. (A). Heatmap and hierarchical clustering of differentially expressed proteins (log_2_ fold change) in JQ1 vs DMSO, IFNγ vs DMSO, and JQ1 + IFNγ vs IFNγ across three temperatures. (B). GSEA reveals pathways regulated in IFNγ-treated and combination-treated cells. Similar analysis was done on proteins grouped based on their expression levels after IFNγ and combination treatment. (C). Scatterplots of protein level changes versus stability changes after IFNγ and combination treatment. The expression change (37 °C protein expression level fold change) is the change of protein abundance (log2 transformed) for each protein at 37 °C in IFNγ and combination-treated cells when compared to the abundance of the same protein at 37 °C in DMSO-treated cells. The stability change (delta fold change of the maximum difference of the protein-level fold changes for each protein across 3 heating temperatures) was also used to segregate proteins into four categories: NN (no change in protein level and no change in thermal stability), NC (no change in protein level but a change in thermal stability), CN (change in protein level but no change in thermal stability), and CC (change in protein level and a change in thermal stability). Some exemplary proteins from each category were labeled, and arrows depict changes in proteins from IFNγ to combination treatment. (D). Heatmap of selected key proteins regulated by JQ1 and/or IFNγ. Data is shown as log_2_ fold change to DMSO.

To determine what molecular mechanisms underlie the immunogenicity-enhancing effects of JQ1, we used a mass spectrometry-based cellular thermal shift assay (MS-CETSA) for a proteome-wide analysis of changes in protein expression and protein-protein interactions in ANRU cells after treatment for 24 h with JQ1 and/or IFNγ (Supplementary Figure S3). Bioinformatics analysis was performed as previously described.^17^ Hierarchical clustering of proteins was performed and grouped based on expression changes at all temperatures across treatments (Figure 2A).

JQ1 alone strongly downregulated CDK6, consistent with its anti-proliferative effects on tumor cells.^19^^,^^20^ In addition, JQ1 strongly decreased expression of IFRD1/IFRD2, both known targets of BRD4 involved in regulating antiviral innate inflammation,^21^ as well as MMP8 and MAP2. In contrast. JQ1 treatment strongly enhanced protein levels of HEXIM1, an established and robust pharmacodynamic marker for target engagement of BETi in cancer cells.^22^ FTL and FTH1 were also amongst the most strongly upregulated proteins by JQ1 treatment, which have been shown to inhibit tumor growth by downregulating c-Myc and promoting ferroptosis.^23^^,^^24^ Of note, JQ1 also increased histone 1 (H1-0 and H1-10) expression, highlighting the role of BET proteins in regulating chromatin homeostasis.

Cells treated with IFNγ alone displayed significant induction of interferon-stimulated genes (ISGs) as well as increased expression of proteins involved in antigen processing and presentation (HLA-A, HLA-C, STAT1, PSMB9, IFI30, and CTSS) and antiviral signaling (OAS2, NMI, IFI35, and GBP1/2). Combined treatment of JQ1 and IFNγ resulted in similar effects on ISGs involved in antigen presentation and antiviral signaling, while downregulating various proteins in a manner similar to JQ1 treatment alone (e.g. MMP8 and IFRD2). Notably, JQ1 enhanced IFNγ-induced expression of HLA-A, HLA-B, HLA-C, β2-migroglobulin (β2M), and STAT1, whereas it suppressed proteins involved in antiviral and immune regulatory pathways like OAS2, NMI, IRFD1, and TRIM21, as well as IFNγ-induced IDO1 expression.

Gene set enrichment analysis (GSEA) revealed that both IFNγ and the combination treatment affected similar pathways involved in antiviral responses, responses to type II IFN, and antigen presentation (Figure 2B). In an effort to better understand the relative contribution of JQ1 in regulating these pathways, GSEA was performed on proteins grouped based on whether JQ1 did not change (IFNγ Up – Combo No change), enhanced (IFNγ Up – Combo Amplified), or suppressed (IFNγ Up – Combo Lowered) their IFNγ-induced expression. The majority of proteins amplified by JQ1 were associated with HLA-I antigen processing and presentation, while those suppressed were involved in antiviral signaling pathways (Supplementary Table S1, Figure 2C). We next analyzed how JQ1 impacts IFNγ-induced changes in the expression and thermal stability of proteins. The use of JQ1 in combination with IFNγ led to a strong stabilization of β2M, an important determinant of efficient peptide presentation on HLA-I molecules,^25^ while solely enhancing protein expression of IFNγ-induced HLA-A, HLA-C, and STAT1, for instance (Figure 2C and D).

To better visualize and summarize these findings, a protein-protein interaction (PPI) network including all CETSA hits was generated, where proteins were annotated based on their expression changes after IFNγ and combination treatment using node and fill color, respectively (Supplementary Figure S4). As previously established, JQ1 modulated the expression and/or stability of IFNγ-induced proteins involved in IFN signaling, antigen processing and presentation, and innate signaling.

Collectively, these data demonstrate that JQ1 exerts pleiotropic effects on IFNγ-responsive pathways, favoring antigen processing and presentation by HLA-I, while restraining components of antiviral and immunoregulatory signaling.

### JQ1 broadly boosts IFN-induced activation of the HLA-I APM while suppressing PD-L1 and IDO

**Figure 3.**
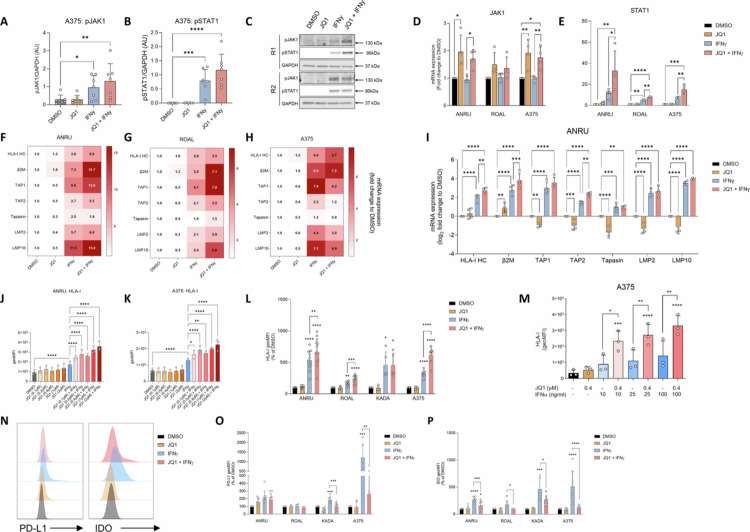
JQ1 broadly boosts IFN-induced activation of the HLA-I APM while suppressing PD-L1 and IDO. (A). Expression of phosphorylated JAK1, (B). STAT1 in A375 cells after 60 min treatment with JQ1 (0.4 μM) and/or IFNγ treatment as assessed by western blot (*n* = 7). (C). Two representative replicates from western blot data displayed in (A and B). (D). Gene expression of JAK1 after 72 h JQ1 (0.4–2 μM) and/or IFNγ treatment in ANRU (*n* = 4), ROAL (*n* = 4), and A375 cells (*n* = 4). (E). Gene expression of STAT1 after 72 h JQ1 (0.4 μM) and/or IFNγ treatment in ANRU (*n* = 4), ROAL (*n* = 3), and A375 cells (*n* = 4). (F). Heatmap of mean expression of genes involved in HLA-I-restricted antigen presentation and immunoproteasome activity upon 72 h treatment with JQ1 (0.4–2 μM) and/or IFNγ in ANRU (*n* = 4), (G). ROAL (*n* = 2) and (H). A375 (*n* = 3). Data is shown as fold change to DMSO. (I). Bar graph displaying expression changes of genes and in conditions shown in (F). Data is displayed as log_2_ fold change to DMSO. (J). Pretreatment for 72 h with JQ1 dose-dependently enhances HLA-I induction by subsequent treatment with IFNγ alone for 24 h in ANRU and (K). A375 cells. *n* = 3/cell line. (L). ANRU, ROAL, KADA, and A375 cells were treated with JQ1 (0.4–2 μM), IFNγ, or a combination of both for 72 h, followed by analysis of HLA-I by flow cytometry. Results are presented as geometric mean fluorescence intensity (geoMFI) as a percentage of control (*n* ≥ 5/cell line). (M). Protein levels of HLA-I after 72 h treatment of A375 cells with JQ1 and/or various concentrations of IFNα, as determined by flow cytometry (*n* = 3). (N). Representative flow cytometry histogram from PD-L1 and IDO stainings in ANRU cells after 72 h JQ1 (0.4 μM) and/or IFNγ treatment, quantified in (O). and (P). respectively in ANRU, ROAL, KADA, and A375 cells. Results are presented as geoMFI as a percentage of DMSO (*n* ≥ 4/cell line).

Building on these findings, we next investigated the impact of JQ1 on IFNγ-mediated regulation of antigen presentation and immunosuppressive pathways in melanoma cells. Under IFNγ stimulation, JQ1 induced only a modest increase in phosphorylation of JAK1 and STAT1, despite markedly increasing *JAK1* and *STAT1* expression (Figure 3A–E). This coincided with potentiated IFNγ-induced upregulation HLA-I APM components, including *HLA-I heavy chain* (*HLA-IHC), β2M, TAP1,* and *TAP2*. JQ1 also enhanced IFNγ-induced expression of Tapasin and the immunoproteasome subunits *LMP2* and *LMP10*, albeit inconsistently between cell lines (Figure 3F–I). This suggests JQ1 primarily primes IFN-related JAK-STAT signaling for downstream transcriptional responses without substantially altering its early phosphorylation events.

Flow cytometry analysis showed that JQ1 dose-dependently enhanced downstream IFNγ-induced HLA-I surface expression but had no effect when used alone (Supplementary Figure S5A). Time-course experiments further revealed that this increase became evident after 48–72 h of combined treatment, indicating that JQ1 sensitizes tumor cells to IFNγ signaling in a time- and dose-dependent manner (Supplementary Figure S5B; Figure 3J–K). This effect was confirmed across multiple melanoma cell lines, except in KADA (Figure 3L). To determine whether this effect extends to type I IFN signaling, we also evaluated the impact of JQ1 on IFNα-induced HLA-I expression. Indeed, JQ1 enhanced IFNα-induced HLA-I surface upregulation in A375 cells (Figure 3M). Importantly, JQ1 displayed opposing effects on other IFNγ-responsive immunosuppressive pathways, where it suppressed PD-L1 and IDO induction (Figure 3N–P).

Together, these results indicate that JQ1 modulates multiple determinants of tumor immunogenicity by sensitizing tumor cells to IFN-mediated signaling and enhancing HLA-I antigen presentation while suppressing immune inhibitory signals like PD-L1 and IDO.

### JQ1 augments recognition of tumor-associated antigens and neo-antigens

**Figure 4.**
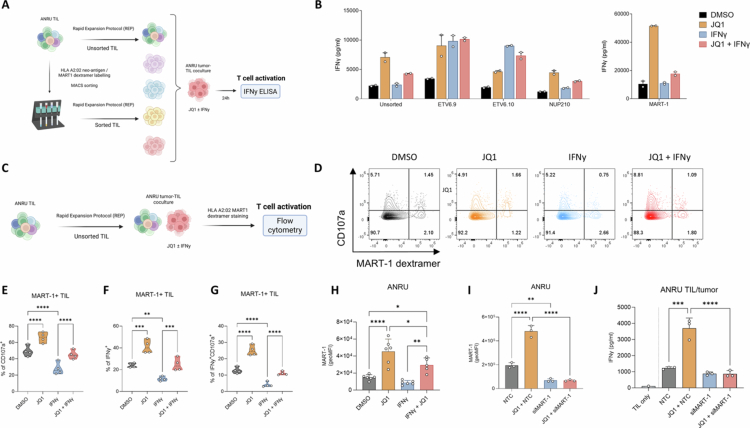
JQ1 augments recognition of tumor-associated antigens and neo-antigens. (A). Experimental design for coculture of sorted antigen-specific TIL with treated autologous ANRU tumor cells. (B). Activation of sorted neoantigen- and MART-1-specific TIL after treatment with JQ1 and/or IFNγ, as assessed by secreted IFNγ (*n* = 2 technical replicates). (C). Experimental design for coculture of stained MART-1-specific TIL with treated ANRU tumor cells. (D). Representative flow cytometry plots from degranulating MART-1-specific TIL when cocultured with treated tumor cells. (E). Frequency of CD107a^+^, (F). IFNγ^+^, and (G). IFNγ^+^CD107a^+^ MART-1-specific TIL upon coculture with treated ANRU tumor cells (*n* = 4). (H). MART-1 protein expression in ANRU after 72 h treatment (*n* = 6). (I). MART-1 protein expression in ANRU after 72 h treatment with JQ1 and/or MART-1 targeted silencing RNAs (*n* = 3). (J). Tumor recognition of JQ1-treated cells by autologous ANRU TIL is abolished in the absence of MART-1 expression (*n* = 3).

Next, we investigated how treatment of tumor cells with JQ1 and/or IFNγ impacts the recognition of epitopes derived from melanoma-associated antigens and neo-epitopes. In an earlier study from our group, several neo-epitopes were predicted and identified as clinically relevant to recognition of the ANRU cell line by autologous TIL.^14^ Custom ordered HLA-A*02:01 multimers carrying the predicted epitopes were used to sort autologous ANRU TIL into subsets of T cells specific for three identified neo-epitopes (ETV6.9, ETV6.10, and NUP10) and for an epitope originating from the shared antigen MART-1 (Figure 4A). Treatment of ANRU tumor cells with JQ1 alone or in combination with IFNγ enhanced recognition of all three neo-epitopes to at least the same extent as treatment with IFNγ alone. For one of these neo-epitopes (NUP210) and the shared melanoma antigen MART-1, recognition improved upon JQ1 pretreatment but not with IFNγ alone (Figure 4B). Furthermore, dextramer stainings were performed to confirm that JQ1 treatment markedly enhanced the recognition of ANRU cells by MART-1-specific TIL and rescued their loss of activation when cultured with IFNγ-pretreated tumors (Figure 4B–G). In line with these data, we found that MART-1 expression in ANRU cells was increased by JQ1 alone or in combination with IFNγ and reduced by IFNγ itself (Figure 4H). These data confirm previous reports demonstrating that prolonged exposure of melanoma cells to T cell-derived cytokines, such as IFNγ, leads to loss of melanoma-associated antigens (MAA) due to dedifferentiation and subsequent loss of recognition by MAA-specific T cells (26). Importantly, silencing of MART-1 in ANRU tumor cells prior to coculture abolished JQ1-induced TIL activation, demonstrating JQ1 primarily enhances activation of autologous CD8^+^ T cells in ANRU by increasing the expression and subsequent presentation of MART-1 (Figure 4I and J). Of note, JQ1 increased the expression of NGFR in ANRU, a marker for melanoma dedifferentiation, while decreasing IFNγ-induced NGFR expression in A375^6^^,^^26^ (Supplementary Figure S6A and B).

Overall, these data indicate that JQ1 enhances tumor antigenicity and promotes activation of both neoantigen- and MART-1-specific CD8⁺ T cells.

### JQ1 suppresses CD4⁺ T cell responses via CTSS-dependent HLA-II regulation

**Figure 5.**
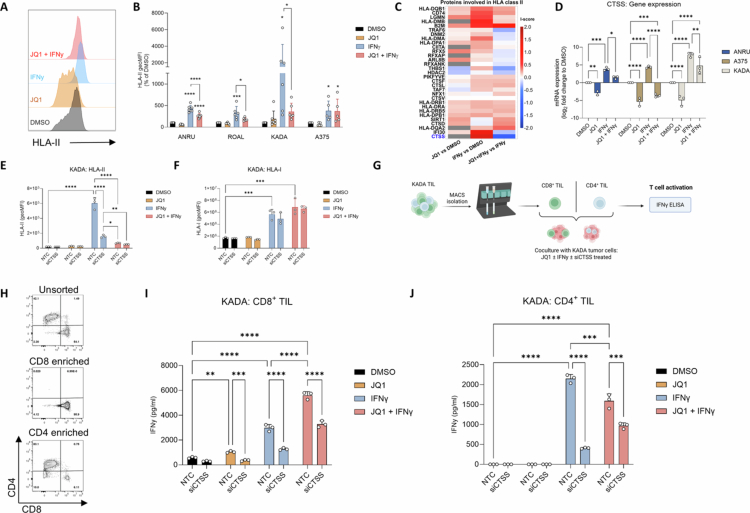
JQ1 selectively potentiates the activation of CD8+ T cells while dampening activation of CD4+ T cells. (A). Representative flow cytometry histogram of HLA-II stainings in ANRU upon JQ1 and/or IFNγ treatment. (B). Surface expression of HLA-II in ANRU, ROAL, KADA, and A375 cells upon treatment. Data is displayed as geoMFI (percentage of DMSO). (C). MS-CETSA analysis of I-scores upon JQ1 and/or IFNγ treatment from proteins involved in HLA-II processing and presentation. Proteins were selected from GO terms that include “MHC class II”. Significantly downregulated protein by JQ1 is labelled in blue (CTSS). (D). CTSS mRNA expression quantified by qPCR after 72 h JQ1, IFNγ or combination treatment in A375 (*n* = 3), ANRU (*n* = 3), and KADA (*n* = 3). Data is displayed as log_2_ fold change to DMSO. (E). HLA-II expression (geoMFI) in treated KADA tumor cells in the presence or absence of CTSS silencing. (F). HLA-I expression (geoMFI) in treated KADA tumor cells in the presence or absence of CTSS silencing. (G). Experimental setup for autologous coculture experiments using MACS sorted CD8^+^ and CD4^+^ KADA TIL. (H). Frequencies of CD8^+^ and CD4^+^ T cells in unsorted KADA TIL or after MACS isolation. (I). Recognition of treated KADA tumor cells by autologous CD8^+^, and (J). CD4^+^ sorted TIL as assessed by IFNγ secretion after 24 h coculture (*n* = 3).

Since JQ1 can boost recognition of tumor cells by autologous CD8^+^ TIL in an antigen-dependent manner, we next wondered how JQ1 might regulate activation of CD4^+^ T cells. JQ1 suppressed baseline and IFNγ-induced HLA-II expression in ANRU, ROAL, and KADA, but not IFNγ-induced HLA-II in A375 cells (Figure 5A and B). Remarkably, proteomic analysis did not reveal any significant changes in expression of key components of the HLA-II APM by JQ1 alone or in combination with IFNγ, with the exception of cathepsin S (CTSS), an enzyme involved in HLA-II antigen processing strongly suppressed by JQ1 (Figure 5C).^27^^,^^28^ CTSS downregulation in ANRU, A375 and KADA cells upon treatment with JQ1 alone or after induction by IFNγ was confirmed at the transcriptional level (Figure 5D). Silencing of CTSS using siRNAs recapitulated the reduction of surface-bound HLA-II by JQ1 without affecting HLA-I levels in KADA (Figure 5E and F). To define the relative contribution of BET inhibition and CTSS targeting to functional T cell responses, CD8⁺ and CD4⁺ KADA TIL were sorted and separately cocultured with treated tumor cells in the presence or absence of CTSS silencing (Figure 5G). Consistent with our earlier findings, JQ1 treatment alone or in combination with IFNγ enhanced CD8⁺ TIL responses while it suppressed IFNγ-induced CD4⁺ T cell activation (Figure 5H–J). Similarly, and to an even greater extent than JQ1, CTSS silencing reduced IFNγ secretion by CD4⁺ T cells. Unexpectedly, however, CTSS targeting also diminished CD8⁺ T cell responses, suggesting that CTSS may influence pathways that contribute to both CD4⁺ and CD8⁺ T cell function.

### JQ1 promotes expansion of CD8^+^ T cells and shows effects across cancer types

To assess if our findings might be applied clinically, BETi-treated tumor cells were used to stimulate the *ex vivo* expansion of autologous TIL in a mixed-lymphocyte tumor cell culture (MLTC), where JQ1- or DMSO- treated ANRU cells were cocultured with autologous TIL (JQ1-TIL and DMSO-TIL, respectively) for ~3 weeks using the established MLTC protocol (18) (Supplementary Figure S7A). JQ1-TIL showed a marked improvement in proliferative capacity as indicated by a higher frequency of ki-67^hi^ T cells and increased yield upon expansion (Supplementary Figure S7B–D). This proliferation was enhanced in CD8^+^ TIL when compared to those in DMSO-TIL (Supplementary Figure 7E). In line with this, JQ1-TIL were enriched for CD8^+^ T cells after expansion compared to DMSO-TIL (Supplementary Figure S7F). These results are in line with our previous findings that JQ1-treatment promotes a tumor phenotype that favors activation of CD8^+^ T cells. Although preliminary, our data suggest that BET inhibition might be therapeutically exploited to improve the selective *ex vivo* expansion of tumor-reactive CD8^+^ T cells.

Finally, to assess whether our findings were restricted to melanoma, four additional cell lines derived from non-small cell lung cancer (NSCL; A549), colorectal cancer (CRC; HCT116), cervical cancer (HeLa), and B cell lymphoma were analyzed after JQ1 and/or IFNγ treatment. In these cell lines, JQ1 treatment boosted IFNγ-induced HLA-I expression, suppressed baseline or IFNγ-induced HLA-II expression, and suppressed IFNγ-induced PD-L1 expression to various extents (Supplementary Figure S8).

Taken together, these findings suggest that BET inhibition promotes a tumor phenotype across multiple cancer types that favors CD8⁺ T cell activation while limiting CD4⁺ T cell responses.

## Discussion

Our study indicates that JQ1 induces pleiotropic molecular changes in melanoma cells that broadly enhance on tumor immunogenicity. This effect is achieved through simultaneous boosting of tumor antigenicity and suppression of various immune inhibitory pathways. Notably, these changes favor tumor recognition by CD8^+^ T cell responses, primarily by elevating components of the HLA-I APM while suppressing PD-L1 and IDO expression. Additionally, we identify a previously unrecognized role for BET proteins in regulating CD4⁺ T cell responses through modulation of tumoral HLA-II expression.

Previous reports have attributed the immunomodulatory effects of BETi to their ability to repress PD-L1 transcription across multiple cancer types, including melanoma.^8-10^^,^^12^ Consistent with this, JQ1 treatment reduced tumor cell PD-L1 expression, potentially due to concurrent suppression of STAT3 by JQ1, as indicated by our MS-CETSA analysis.^29^ However, this reduction did not correlate with enhanced recognition by autologous T cells. Therefore, the contribution of JQ1-mediated suppression of PD-L1 and IDO to human T cell activation in this context remains unclear and warrants further investigation.

Recent work suggests that immunosuppressive pathways, such as PD-L1, IDO and regulatory T cell (Treg) induction, are primarily driven by IFNγ-producing T cells rather than tumor-intrinsic mechanisms.^30^ BETi might therefore act synergistically with IFNγ to amplify the immunogenicity-enhancing effects of IFNγ, such as upregulation of HLA-I APM components, while simultaneously limiting IFNγ-induced immunosuppressive mechanisms. Approaches targeting such immunological checkpoints, including BET inhibition, could potentially boost antitumor immunity in both “hot” tumors with preexisting T cell infiltration that are sensitive to PD-L1 blockade, and “cold” tumors, where enhanced activation of the HLA-I APM could initiate T cell recruitment. Supporting this concept, CRISPR-Cas9 loss-of-function screens have identified that the HLA-I APM and IFNγ signaling pathways are key determinants of resistance to T cell-based immunotherapy.^31^ Our data indicate that BETi precisely affects these pathways, highlighting their potential to improve the efficacy of T cell-based therapies. Combined with their known anti-proliferative activity, BETi may therefore represent promising anti-cancer therapeutics that promote tumor eradication through multiple mechanisms.

Modulation of BET-dependent transcriptional programs has been implicated in controlling the expression of differentiation antigens in melanoma cell lines and target a gene regulatory network conferring immunotherapeutic resistance in melanoma cells, particularly in mesenchymal-like phenotypes.^32^^,^^33^ We show that JQ1 induces NGFR expression either alone or in combination with IFNγ in ANRU cells, which suggests that it promotes tumor dedifferentiation, associated with loss of melanoma antigens and resistance against T cell killing.^6^^,^^34^ However, NGFR induction by JQ1 coincided with increased expression of the melanoma differentiation antigen MART-1 and strongly improved recognition by autologous MART-1-specific TIL. This suggests that BET proteins can regulate phenotype switching in a manner that preserves or even improves tumor antigenicity, and points to a more nuanced role of BET proteins in coordinating differentiation and functional immune outcomes.

A key observation in this study is the opposing role of JQ1 in promoting antigen presentation to CD8^+^ T cells and in suppressing HLA-II expression and recognition by CD4^+^ T cells. MS-CETSA analysis suggests this effect is mediated through suppression of CTSS, since other proteins related to the HLA-II APM were not affected. Interestingly, in murine models of B cell lymphoma, CTSS targeting inhibited tumor outgrowth by limiting pro-tumoral CD4^+^ T cell activation and simultaneously broadening antigen presentation by MHC-I molecules. Although knockdown of CTSS in our model was associated with reduced CD8⁺ T cell responses, this may reflect the diversification of the antigenic repertoire presented on HLA-I, which could prevent the activation of an already skewed population of antigen-specific TIL.

The role of CD4^+^ T cells in solid tumors remains highly context-dependent: some reports suggest CD4^+^ T cell engagement is critical in mediating clinical response to immunotherapy, while others claim immune suppressive CD4^+^ TIL, such as CD4^+^ Tregs, actively participate in immune escape.^35-38^ Our findings suggest that JQ1 may tilt this balance toward antitumor immunity by selectively enhancing CD8⁺ T cell responses while restraining potentially suppressive CD4⁺ TIL activity, in line with murine CRC models where BET inhibition promoted CD8⁺ T cell activation and reduced tumor-infiltration of CD4⁺ Tregs.^39^ These findings underscore the importance of considering CD4^+^ T cell modulation when developing BET-targeting approaches as an anti-cancer therapy.

To explore the clinical relevance of our findings, we demonstrated that coculture of JQ1-treated tumor cells with autologous TIL in an *ex vivo* MLTC system improved the expansion and proliferative capacity of tumor-reactive CD8⁺ T cells. This effect may be partly due to suppression of tumor-derived IDO, which is known to limit T cell expansion in this system.^40^ Although preliminary, our data suggest BET inhibition could be exploited for the selective *ex vivo* expansion of tumor-reactive CD8^+^ T cells for adoptive T cell therapies.

This study has several limitations that should be considered when interpreting its findings. First, our experiments were performed essentially *in vitro* and *ex vivo* using tumor cell lines and coculture systems with autologous TIL, which do not fully capture the complexity of the tumor microenvironment. Validation in appropriate *in vivo* models will be required to determine whether the immunogenic effects of BETi observed here translate into improved anti-tumor immune responses. Second, our study focused exclusively on JQ1 as a BETi, known as a well-characterized tool to perturb BET protein function. Additional studies using structurally distinct BETi or alternative BET-targeting strategies will be important to determine the generalizability and therapeutic relevance of these findings.

Overall, this study provides novel insights into the wide-ranging impact of BETi on key molecular pathways that govern tumor immunogenicity in melanoma and other cancers, and how these BETi-induced immunogenicity-enhancing effects affect recognition of various tumor antigens by autologous CD8^+^ T cells. Ultimately, these results can guide future research to explore whether BETi might be clinically applied to improve the efficacy of cancer immunotherapies.

## Supplementary Material

Supplementary Material Supplementary Figure S6.TIF

Supplementary Material Supplementary Figure S8.TIF

Supplementary Material Supplementary Table S1.xlsx

Supplementary Material Supplementary Figure S4.TIF

Supplementary Material Supplementary Figure S3.TIF

Supplementary Material Supplementary Figure S2.TIF

Supplementary Material Supplementary Figure S1.TIF

Supplementary Material Supplementary Figure S5.TIF

Supplementary Material Supplementary Figure S7.TIF

Supplementary Material Supplementary.docx

## Data Availability

All data from this study are available upon reasonable request. Additional raw data and analysis from the CETSA experiment are available at: https://doi.org/10.6084/m9.figshare.31964637.
