# Supplementary figures and images for: Pleiotropic effects of BET inhibition broadly boost tumor immunogenicity to CD8+ T cells
Source: Oncoimmunology. 2026 Apr 29;15(1):2658916. doi: 10.1080/2162402X.2026.2658916 (PMC13134409; doi:10.1080/2162402X.2026.2658916)

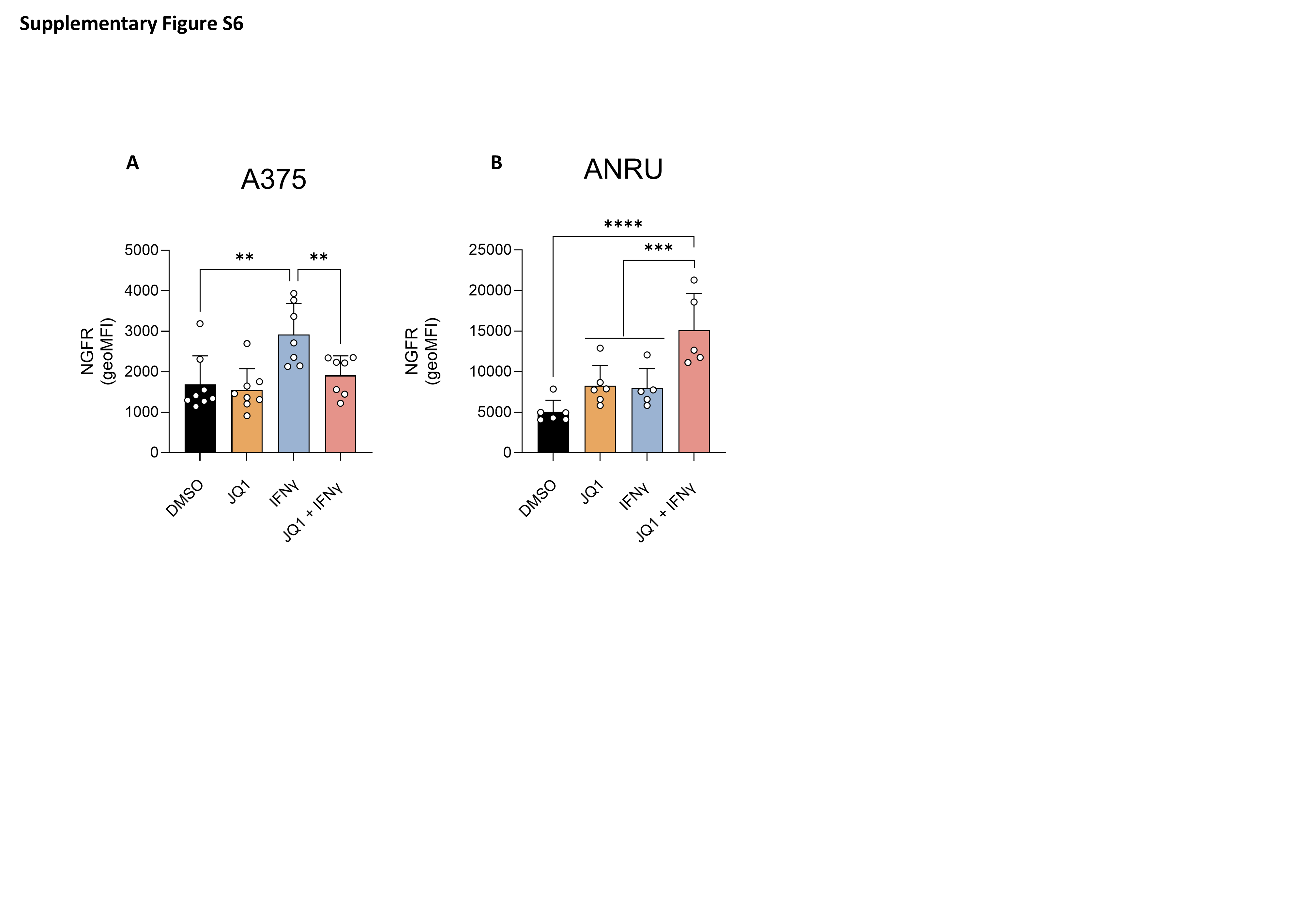

Supplement: Supplementary Material — Supplementary Figure S6.TIF [file KONI_A_2658916_SM9949.tif]

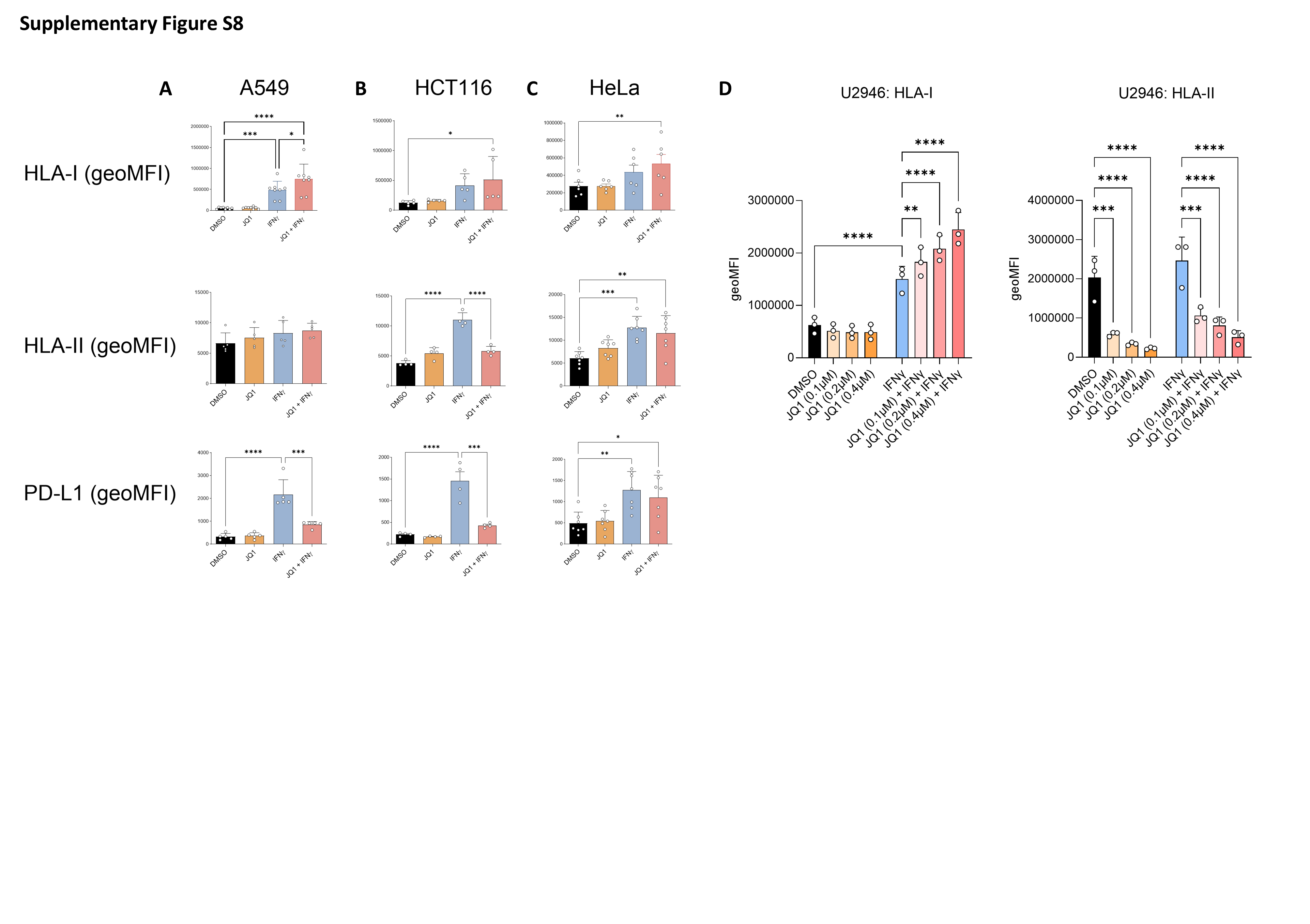

Supplement: Supplementary Material — Supplementary Figure S8.TIF [file KONI_A_2658916_SM9267.tif]

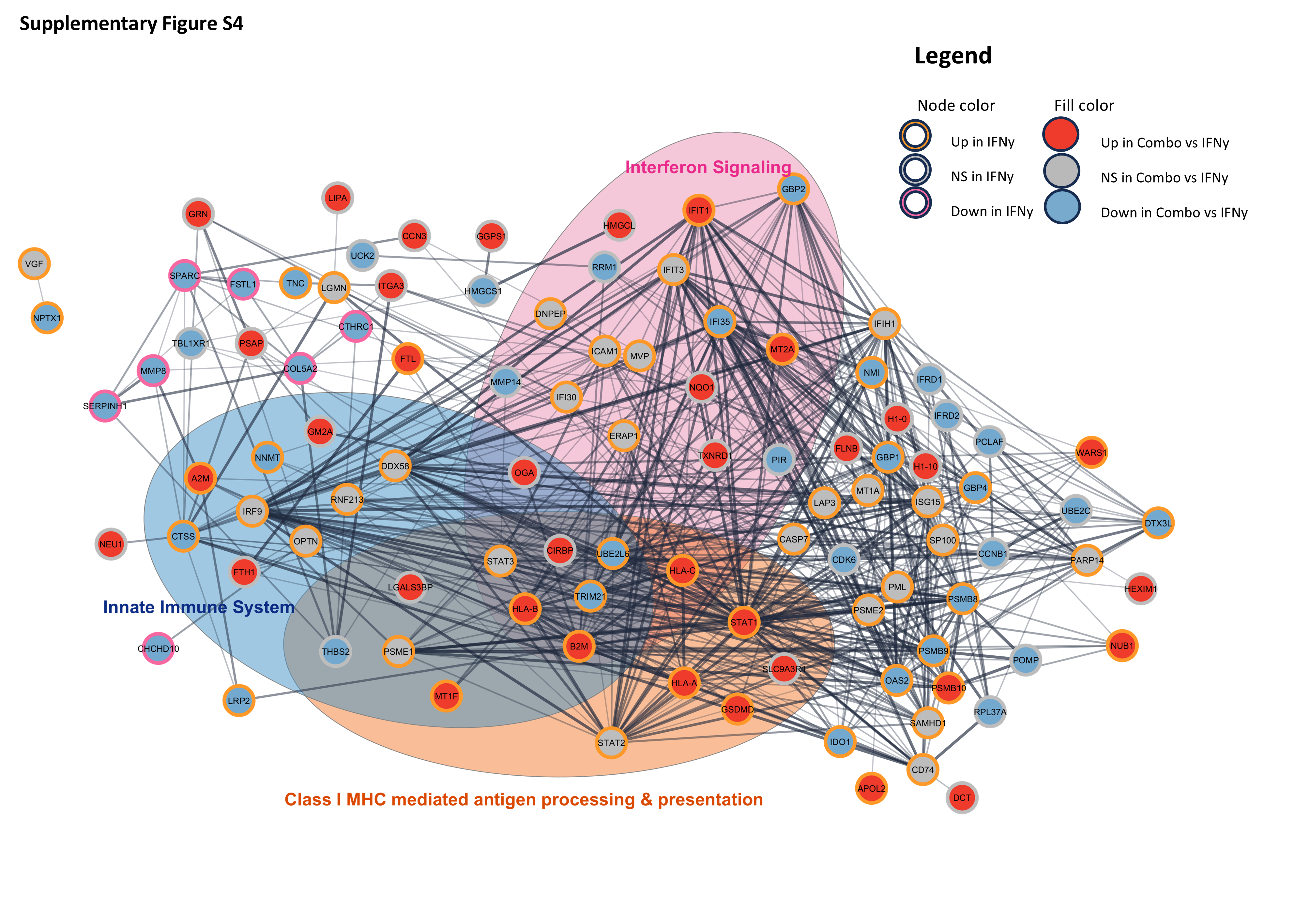

Supplement: Supplementary Material — Supplementary Figure S4.TIF [file KONI_A_2658916_SM0499.tif]

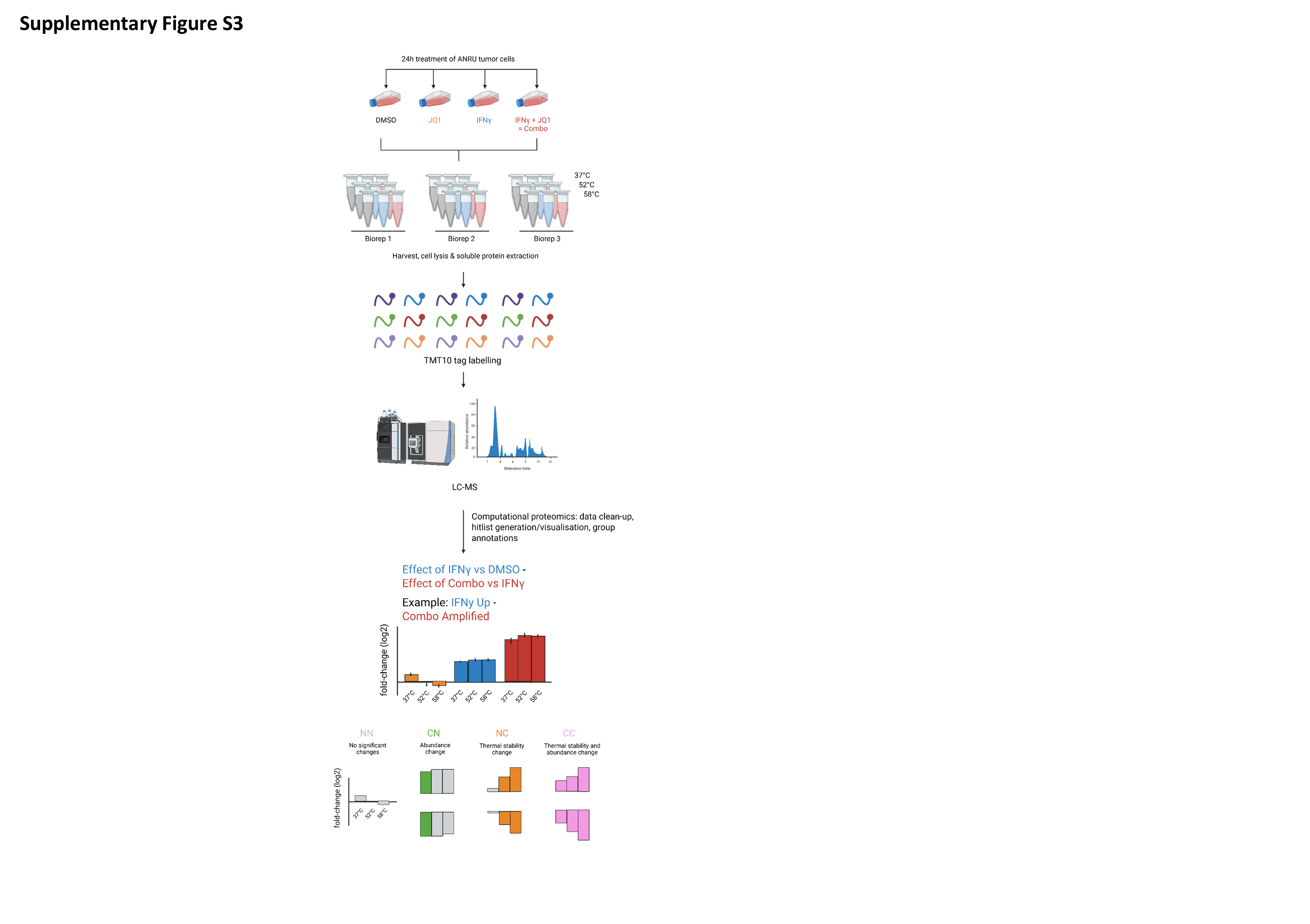

Supplement: Supplementary Material — Supplementary Figure S3.TIF [file KONI_A_2658916_SM0800.tif]

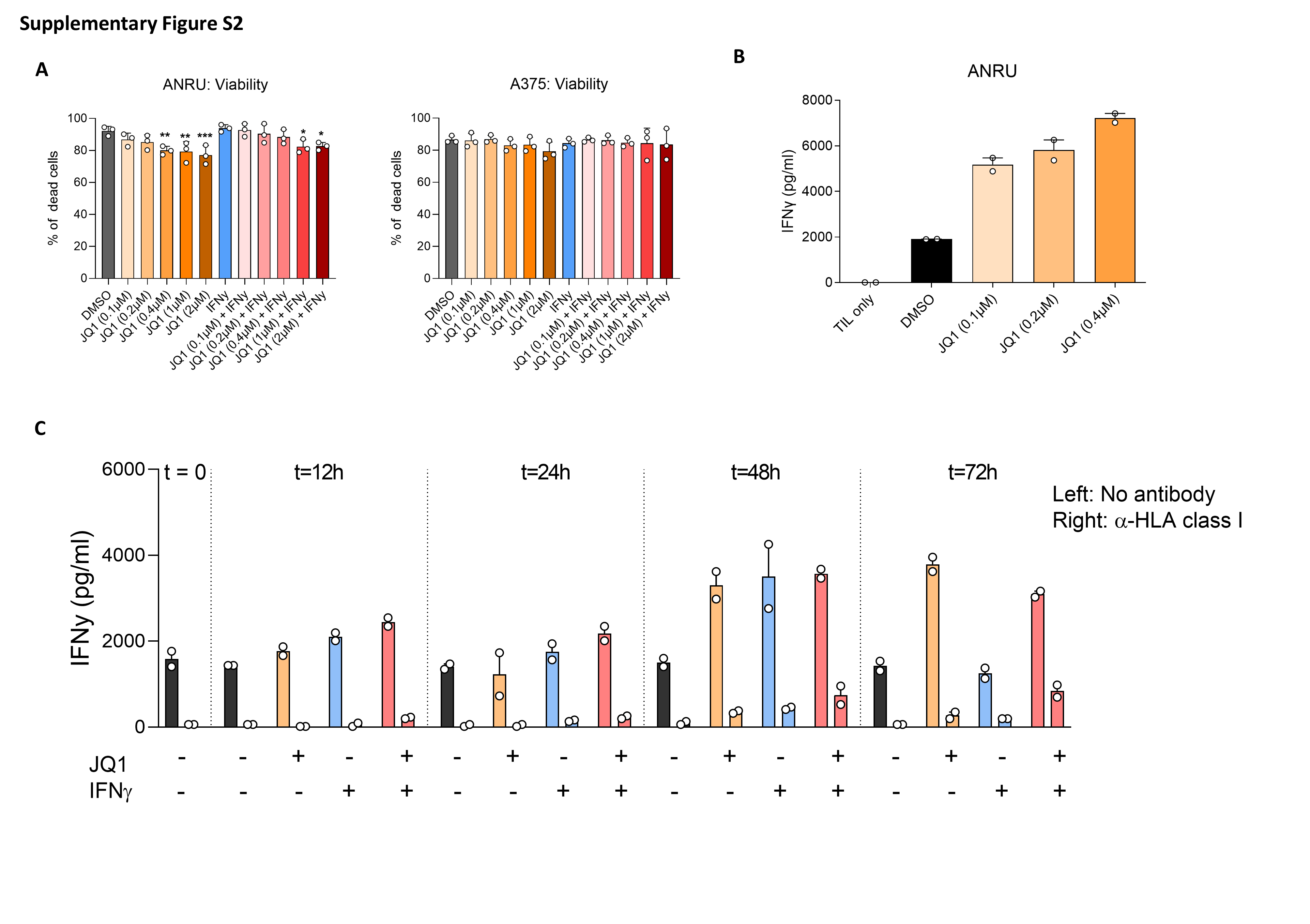

Supplement: Supplementary Material — Supplementary Figure S2.TIF [file KONI_A_2658916_SM1035.tif]

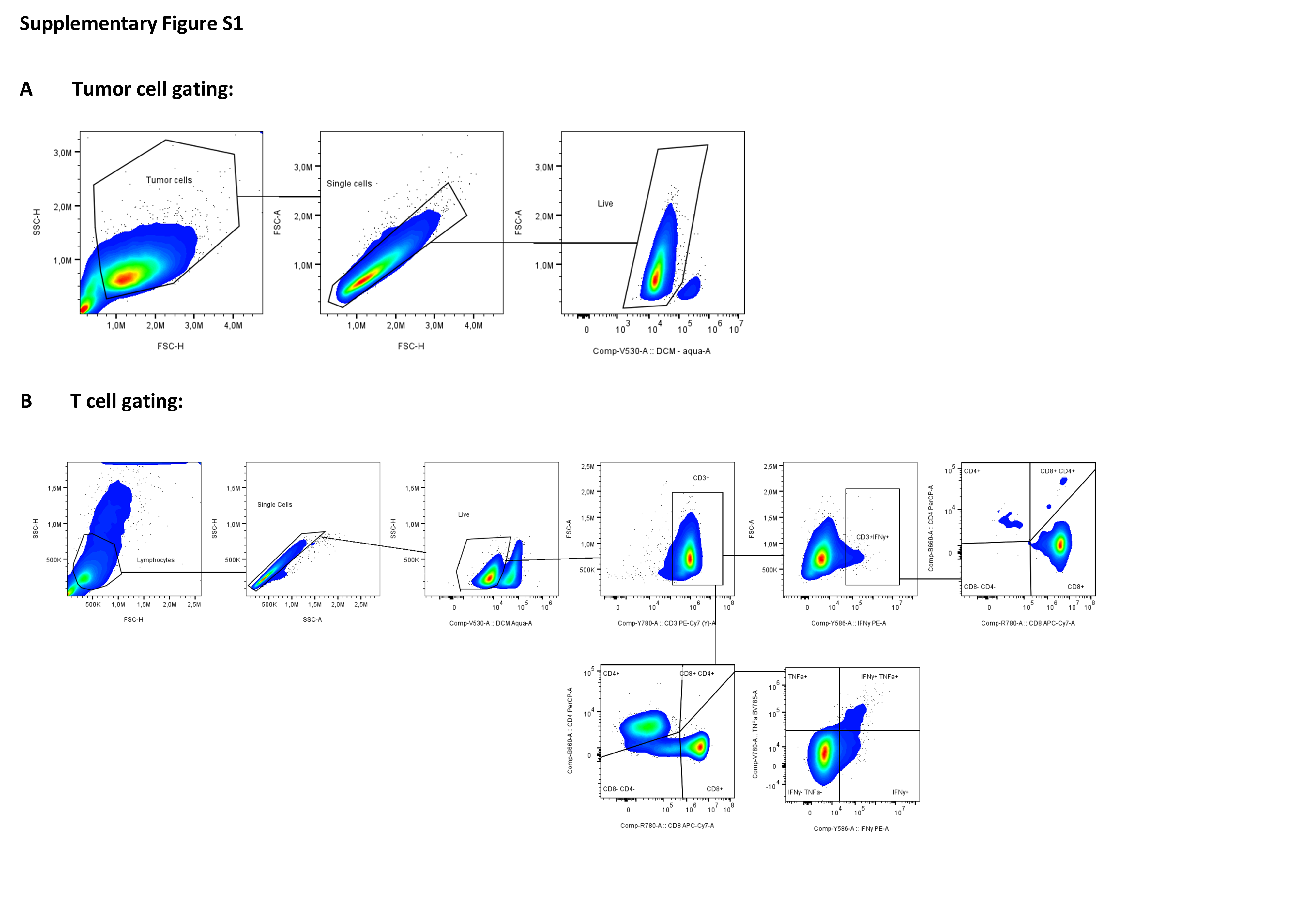

Supplement: Supplementary Material — Supplementary Figure S1.TIF [file KONI_A_2658916_SM1228.tif]

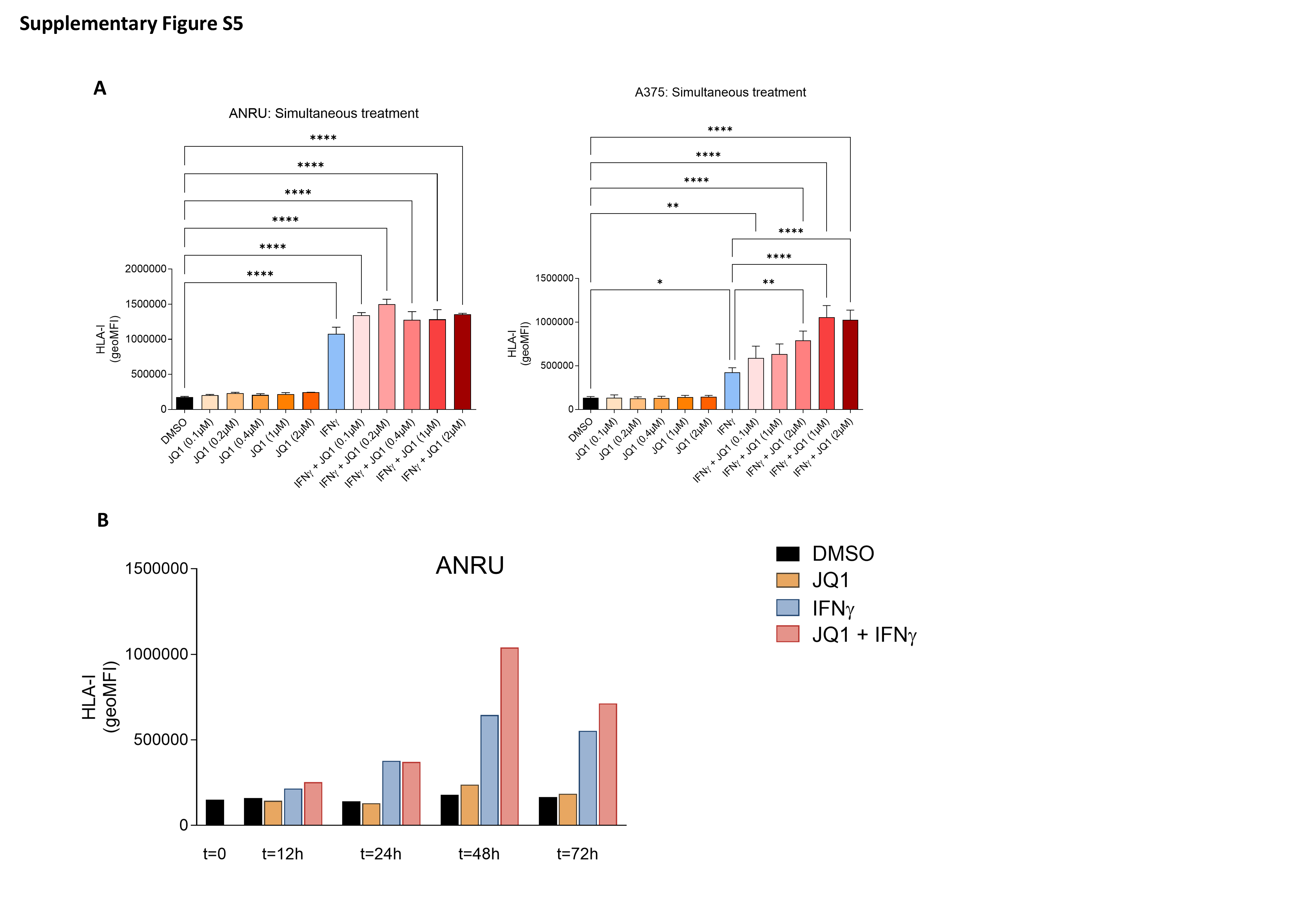

Supplement: Supplementary Material — Supplementary Figure S5.TIF [file KONI_A_2658916_SM0192.tif]

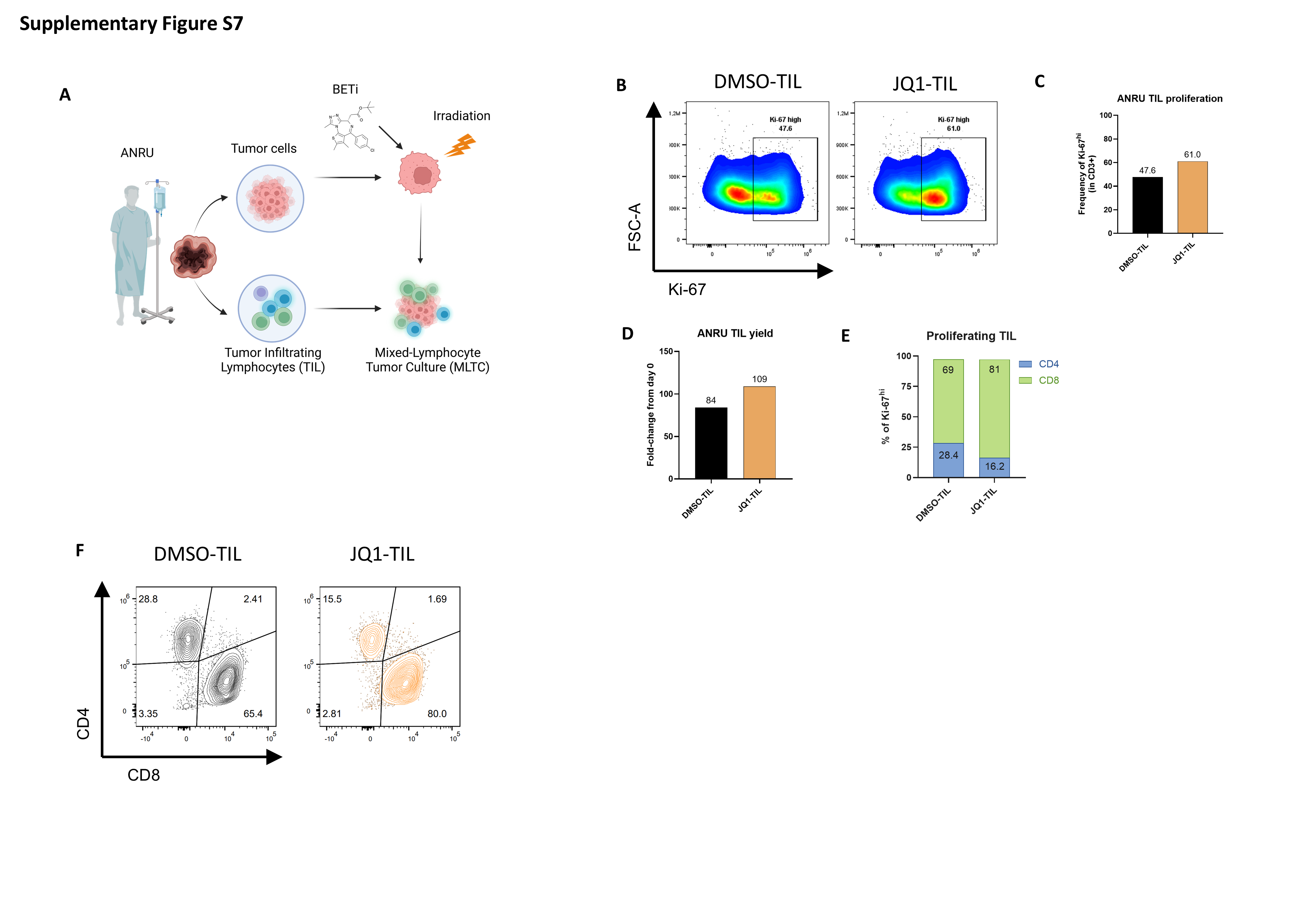

Supplement: Supplementary Material — Supplementary Figure S7.TIF [file KONI_A_2658916_SM9608.tif]
