# Supplementary material for: Pleiotropic effects of BET inhibition broadly boost tumor immunogenicity to CD8+ T cells
Source: Oncoimmunology. 2026 Apr 29;15(1):2658916. doi: 10.1080/2162402X.2026.2658916 (PMC13134409; doi:10.1080/2162402X.2026.2658916)
Supplement: Supplementary Material — Supplementary.docx [file KONI_A_2658916_SM6493.docx]

**Supplementary Table S1. Gene enrichment analysis of JQ1/IFNγ hits**

**Supplementary Figure S1.** Flow cytometry gating strategy for A. tumor cells and B. T cells.

**Supplementary Figure S2. Dose- and time-kinetics experiments for JQ1 treatment. A.** Frequency of dead cells upon JQ1 and/or IFNγ treatment in ANRU and KADA assessed by flow cytometry. **B.** Dose-dependent sensitization of ANRU tumor cells for recognition by autologous TIL after 72h of JQ1 treatment, (n=2 technical replicates) **C.** Time kinetics experiment for IFNγ production by ANRU TIL upon coculture with JQ1 and/or IFNγ treated ANRU tumor cells. (n=2 technical replicates).

**Supplementary Figure S3.** Experimental setup for MS-CETSA experiment.

**Supplementary Figure S4.** Protein-protein interaction network generated from MS-CETSA hits. Single nodes were excluded for this analysis. Node colors depict changes in protein expression/stability after IFNγ-treatment compared to DMSO, and fill color indicates protein changes after combination treatment compared to IFNγ-treated cells. Network was generated using the Cytoscape app.

**Supplementary Figure S5. Dose- and time-kinetics experiment of HLA-I induction by JQ1. A.** Treatment for 72h with JQ1 dose-dependently enhances HLA-I induction when combined with IFNγ (n=2) **B.** Time-dependent sensitization of JQ1 on ANRU tumor cells for HLA-I induction by IFNγ (n=1).

**Supplementary Figure S6. A.** NGFR expression in A375 and **B.** ANRU cells after 72h JQ1, IFNγ or JQ1/IFNγ treatment (n ≥ 6).

**Supplementary Figure S7. Expansion of tumor-reactive T cells by JQ1-treated tumor cells in MLTC**. **A.** Schematic overview of MLTC experiment. **B, C.** Increased proliferation of JQ1-TIL as assessed by higher frequency of ki-67^hi^ cells and **D.** overall yield. **E.** Proliferation of JQ1-TIL is skewed to CD8+ T cells compared to DMSO-TIL. **F.** Flow cytometry analysis of CD8^+^ and CD4^+^ phenotypes after MLTC. JQ1-TIL are enriched in CD8+ T cells when compared to DMSO-TIL.

**Supplementary Figure S8. A-C** HLA-I, HLA-II and PD-L1 expression in various solid tumor cell lines. (n≥4). **D.** HLA-I and HLA-II expression in U2946 cells after JQ1, IFNγ or combination treatment (n = 3 biological replicates).
